# Improving the quality of care for patients with or at risk of atrial fibrillation: an improvement initiative in UK general practices

**DOI:** 10.1136/openhrt-2019-001086

**Published:** 2019-10-15

**Authors:** Yewande Adeleke, Dionne Matthew, Bradley Porter, Thomas Woodcock, Jayne Yap, Sophia Hashmy, Ammu Mathew, Ron Grant, Agnes Kaba, Brigitte Unger-Graeber, Sadia Khan, Derek Bell, Martin R Cowie

**Affiliations:** 1 Department of Primary Care and Public Health, Imperial College London, Chelsea and Westminster Hospital, NIHR Collaboration for Leadership in Applied Health Research and Care for Northwest London, London, UK; 2 Chelsea and Westminster Hospital, Chelsea and Westminster Hospital NHS Foundation Trust, London, UK; 3 Cardiology Department, Guy's and St Thomas' NHS Foundation Trust, London, UK; 4 St Thomas' Hospital, King's College London, London, UK; 5 Clinical Quality, Performance and Technology, Government of Singapore Ministry of Health, Outram, Singapore; 6 North West London Clinical Commissioning Groups, London, UK; 7 Cardiology Department, West Middlesex University Hospital, Chelsea and Westminster Hospital NHS Foundation Trust, London, UK; 8 Upbeat Heart Prevention and Support Group, London, UK; 9 Chiswick Health Practice, London, UK; 10 National Heart and Lung Institute, Imperial College London, London, UK

**Keywords:** atrial fibrillation, electrocardiography, quality improvement, risk stratification, anticoagulation

## Abstract

**Objective:**

Atrial fibrillation (AF) is a growing problem internationally and a recognised cause of cardiovascular morbidity and mortality. The London borough of Hounslow has a lower than expected prevalence of AF, suggesting poor detection and associated undertreatment. To improve AF diagnosis and management, a quality improvement (QI) initiative was set up in 48 general practices in Hounslow. We aimed to study whether there was evidence of a change in AF diagnosis and management in Hounslow following implementation of interventions in this QI initiative.

**Methods:**

Using the general practice information system (SystmOne), data were retrospectively collected for 415 626 patients, who were actively registered at a Hounslow practice between 1 January 2011 and 31 August 2018. Process, outcome and balancing measures were analysed using statistical process control and interrupted time series regression methods. The baseline period was from 1 January 2011 to 30 September 2014 and the intervention period was from 1 October 2014 to 31 August 2018.

**Results:**

When comparing the baseline to the intervention period, (1) the rate of new AF diagnoses increased by 27% (relative risk 1.27; 95% CI 1.05 to 1.52; p<0.01); (2) ECG tests done for patients aged 60 and above increased; (3) CHA_2_DS_2_-VASc and HAS-BLED risk assessments within 30 days of AF diagnosis increased from 1.7% to 19% and 0.2% to 8.1%, respectively; (4) among those at higher risk of stroke, anticoagulation prescription within 30 days of AF diagnosis increased from 31% to 63% while prescription of antiplatelet monotherapy within the same time period decreased from 17% to 7.1%; and (5) average CHA_2_DS_2_-VASc and HAS-BLED risk scores did not change.

**Conclusion:**

Implementation of interventions in the Hounslow QI initiative coincided with improved AF diagnosis and management. Areas with perceived underdetection of AF should consider similar interventions and methodology.

**Key questions**

**What is already known about this subject?**

- Atrial fibrillation (AF) is a growing problem internationally and a recognised cause of cardiovascular morbidity and mortality. Despite published guidance, it is often undetected or undertreated in practice. Handheld ECG devices may facilitate screening of large numbers of individuals at risk of having this condition. Education of healthcare professionals and patients has been shown to increase appropriate oral anticoagulant prescription.

**What does this study add?**

- This study found that use of quality improvement methodology to implement evidence-based interventions, including handheld ECG devices, may help improve AF diagnosis and management within the primary care setting.

**How might this impact on clinical practice?**

- These findings suggest that healthcare professionals working in areas with evidenced underdetection and undertreatment of AF should consider implementing similar interventions using quality improvement methodology.

## Introduction

Atrial fibrillation (AF) is a common heart arrhythmia affecting 34 million people worldwide^1^ and is associated with increased risk of cardiovascular morbidity and mortality, including ischaemic stroke.^2 3^ The European Society of Cardiology (ESC) and the National Institute for Health and Care Excellence (NICE) have published guidelines for management of AF.^4 5^ Despite this, AF is often undetected and undertreated.^6^

A multitude of factors contribute to poor implementation of AF guideline recommendations by healthcare professionals (HCP).^7–9^ To encourage implementation, many strategies have been employed. For instance, handheld ECG devices have been identified as feasible and efficient screening options for people at risk of developing AF.^10 11^ A systematic review identified that HCP and patient education significantly increased appropriate oral anticoagulant prescription, whereas computerised decision support tools and medication reviews did not.^12^ However, another study has shown that systematic identification and risk stratification of patients using electronic tools in conjunction with case note reviews and anticoagulation assessment clinics have improved AF diagnosis and management.^13^

In the UK, AF affects 1.2 million people^14^ and accounts for 1.0% of the total National Health Service (NHS) expenditure.^15^ Hounslow Clinical Commissioning Group (CCG) is one of eight CCGs in Northwest London (NWL), and comprises 48 general practices.^16^ In 2014, up to 2492 out of 292 220 patients registered with a general practice in Hounslow borough had a diagnosis of AF, giving an observed prevalence of 0.9%,^17^ compared with an expected prevalence of 1.6%.^18^ This suggests nearly 2000 people with undiagnosed and untreated AF. In the same year, 98.8% of patients diagnosed with AF were assessed for risk of stroke and 84.8% of patients diagnosed with AF with a CHADS_2_ score greater than 1 were prescribed anticoagulants.^17^ Despite these high proportions, there was a lack of information on timeliness of care from point of diagnosis. Furthermore, Hounslow’s population aged over 65 is projected to increase by 18% by 2020,^19^ this may have major implications for health service provision within the borough, as AF prevalence and incidence increases with age.^1^

In 2014, the Hounslow Quality Improvement (QI) team, supported by the National Institute for Health Research Collaboration for Leadership in Applied Health Research and Care NWL (CLAHRC NWL), began a QI initiative aiming to improve diagnosis and management of care for patients with or at risk of AF in Hounslow. This study aimed to evaluate the extent to which these aims were achieved.

## Methods

The intervention was a QI initiative comprising education and specialist support for general practitioners (GP) in diagnosis and management of AF, alongside provision of KardiaMobile (AliveCor) handheld ECG devices to facilitate diagnosis.

### Study population

The study population comprised patients registered at a general practice in Hounslow CCG prior to 31 August 2018, and who were still on the register by 31 August 2018 or ceased to be registered at some time after 1 January 2011. The latter is referred to as ‘deduction’ and occurs either through death or by deregistering, for example, moving out of Hounslow.

### Data extraction

Pseudonymised data, in the form of Read-coded events, were retrospectively extracted from the SystmOne clinical general practice database on 10 September 2018. Data were extracted for the following events: registration with a GP practice, deduction from a practice list, ECG screening, diagnosis of AF, risk scoring for stroke (CHA_2_DS_2_-VASc) and bleeding (HAS-BLED) and prescription of anticoagulation. The Read code and event date were extracted, along with the age, gender and ethnicity of the patient, risk scores, and prescription drug, dose and duration. The data were cleaned to remove patients with missing or non-sensical registration dates (n=3281), non-sensical deduction dates (n=523), deduction dates preceding registration dates (n=405) and non-sensical registration status (n=66), some of these were duplicate patient records. A senior GP, with direct clinical responsibility for these patients, compared a sample of extracted data with patient records on SystmOne for data validation purposes. Data on AF diagnoses prior to 2011 could not be obtained from SystmOne. Instead, prevalence data were retrospectively collected from the Quality and Outcomes Framework (QOF) database for each year from 2011 to 2018.

### Measures

The primary outcome of our investigation was the change in rate of AF diagnosis in Hounslow over time. The following outcome measures were assessed across the study population monthly: (1) proportion of patients with a new or pre-existing diagnosis of AF (prevalence); (2) number of patients newly diagnosed with AF; and (3) rate of new AF diagnosis. AF diagnoses were counted for all patients diagnosed with AF (including patients with an AF diagnosis alongside other comorbidities), irrespective of method of diagnosis such as 12-lead ECG, ambulatory ECG monitoring and KardiaMobile handheld ECG. There was no Read code specifically for the KardiaMobile device.

A ‘risk register’ of patients at high risk of AF was not available retrospectively, so overall numbers of ECG tests done in patients aged 60 and above were used as a proxy measure. Process measures after AF diagnosis measured four recommended elements of care: the proportion of patients who received a CHA_2_DS_2_-VASc risk assessment; proportion of patients who received a HAS-BLED risk assessment; proportion of indicated patients (ie, patients diagnosed with AF who have a CHA_2_DS_2_-VASc score of greater than or equal to 1 for males or greater than or equal to 2 for females) prescribed anticoagulation drug therapy; and proportion of indicated patients prescribed antiplatelet monotherapy. To understand the timeliness of the care provided we measured each proportion at 30, 90 and 180 days from AF diagnosis, along with the proportion who received the element in question at any point following diagnosis.

To understand whether efforts to increase AF diagnosis through screening inadvertently reached a subpopulation less at risk of stroke or bleeding than others, we measured the average CHA_2_DS_2_-VASc and HAS-BLED scores among patients newly diagnosed with AF. These measures acted as ‘balancing measures’.^20^

### Statistical analysis

Demographics of the study population, and the subpopulation with an AF diagnosis, were analysed as distributions of categorical variables age band, sex and ethnicity. Study population demographics were also evaluated at three time points: 31 December 2011; 31 December 2014; and 31 December 2017. Categorical variables are reported as total numbers and percentages and continuous variables as medians±IQR.

The baseline period, before the QI initiative began, was 1 January 2011 to 30 September 2014, and the intervention period was 1 October 2014 to 31 August 2018. Statistical process control (SPC) charts including C charts, P charts and $\bar{X}$ bar S control charts were created to understand the variation in the outcome, process and balancing measures over time and monitor the effect of changes.^21^ Established rules were used to determine whether variation was down to chance (online supplementary file 2).^21^ SPC analysis was performed using Microsoft Excel (V.1808).

10.1136/openhrt-2019-001086.supp2 Supplementary data

To assess the rate of new AF diagnosis, an interrupted time series (ITS) regression was conducted.^22^ A level change model was selected, based on evidence that opportunistic screening using ECGs increases rates of AF diagnosis.^23^ An age and sex-standardised population was used to adjust for changes to population structures over time. P values <0.05 were deemed statistically significant. Quasi-Poisson regression was used to account for overdispersion in the data. The independent variables were time in months and the intervention. R statistical software (V.3.5.0) was used for the ITS analysis.

Pearson’s χ^2^ test was used to compare whether indicated patients who have a HAS-BLED score were more likely to be anticoagulated than those who did not have a HAS-BLED score.

### QI initiative

The QI team consisted of a consultant cardiologist, GP commissioner, cardiology nurses, project manager, CLAHRC NWL QI advisors and a patient with lived experience of AF. A systematic approach was used to guide evidence translation into practice,^24^ making use of a range of improvement tools and methods, including the Model for Improvement, Stakeholder Mapping, Process Mapping, Action Effect Diagram^25^ and Plan-Do-Study-Act cycles. The intervention period was split into three phases (see online supplementary file 1 for the corresponding timeline).

10.1136/openhrt-2019-001086.supp1 Supplementary data

### Interventions

#### Phase I (October 2014 to January 2016)

An educational launch event was designed to improve HCPs’ knowledge of AF diagnosis and management, and to highlight best practice. At this event, presentations were delivered by the QI team to representatives from all Hounslow general practices. The project manager demonstrated the KardiaMobile ECG device and how to record findings in SystmOne. Following this, the project manager made ad hoc general practice visits, which comprised education of GPs, practice nurses and health visitors on the importance of early detection of AF, available treatment options, how to use the handheld ECG device, the aim of the QI initiative and its measures of success. These visits were tailored to the practice, and individual need, and lasted approximately 1 hour. In addition, the team attended Hounslow CCG network meetings to discuss the QI initiative with representatives from Hounslow general practices.

#### Phase II (February 2016 to June 2016)

Two handheld ECG devices and two mobile devices were distributed to each of the five pilot general practices to facilitate screening for AF within GP consultations, diabetes clinics, NHS health checks, influenza jab clinics, and so on. Mobile devices were used to encourage communication between HCPs and the QI team, for instance, to address queries about abnormal readings, or about the devices. A guide for HCPs on how to use the handheld ECG devices and mobile devices was created by the project manager using Microsoft PowerPoint.

An AF at-risk register was developed by the consultant cardiologist and the lead GP. The inclusion criteria for the at-risk register were patients aged 60 and above or on at least one of four disease registers: coronary heart disease, chronic obstructive pulmonary disease, obstructive sleep apnoea or hypertension. These disease registers were chosen in view of the community services for diabetes and heart failure already in existence in the borough, and that these conditions alone gave an at-risk register of more than 50 000 people. A SystmOne AF clinical template was adapted from Nene CCG to encourage evidence-based practice in Hounslow and to upskill GPs on AF diagnosis and management. Patients who met the AF at-risk register criteria were flagged to GPs by a patient status alert linking to the clinical template.

#### Phase III (July 2016 to March 2017)

Each of the 43 remaining general practices received two handheld ECG devices, two mobile devices and an AF QOF data report for their practice. The same set-up process was followed as in phase II.

The QI team collaborated with HCPs and patients to coproduce eleven 5 min long ‘electronic postcards’ (videos, one of which was in Punjabi) to improve patient understanding of AF during and after consultation. These videos consist of members of the cardiology team explaining their role and giving information about AF, its causes, symptoms, diagnosis, treatment options for rate and rhythm control and common side effects, the referral pathway, follow-up process, the ambulatory care pathway and patient support groups. Two of the videos feature patients living with AF describing their experience of the condition, how it has affected their quality of life and the lifestyle changes they made to manage their AF.

For all Hounslow general practices, a cardiology nurse reviewed AF registers, focusing on exception-reported patients and patients on aspirin, and ran face-to-face anticoagulation clinics.

The Standards for Quality Improvement Reporting Excellence 2.0 guidelines were used as a framework for reporting findings from this QI initiative.^26^

## Results

### Characteristics of study population

Of 48 Hounslow general practices, data were available from 47 using the SystmOne software, but were not available from one practice that used a different clinical software (Egton Medical Information Systems). The study population consisted of 415 626 patients registered at these practices, of which 3007 had an AF diagnosis at any time point from 1 January 2011 to 10 September 2018 (table 1). The study population had a median age of 36±27 years, whereas patients with an AF diagnosis were older with a median age of 77±17 years. AF diagnosis was most common in patients aged 80–89 years old (30%) and ethnically white (65%); known risk factors for AF (table 1). Patient demographics remained similar over the duration of the study (online supplementary file 3).

10.1136/openhrt-2019-001086.supp3 Supplementary data

**Table 1:** Demographic characteristics of the Hounslow study population and the subpopulation stratified by AF diagnosis

Characteristic	Study population (%)	AF subpopulation (%)
Registration status		
Current	276 239 (66.5)	1728 (57.5)
Deceased, deducted	7763 (1.9)	520 (17.3)
Deducted	131 624 (31.7)	759 (25.2)
Sex		
Female	194 019 (46.7)	1363 (45.3)
Male	221 482 (53.3)	1642 (54.6)
Other/unknown	125 (0.0)	2 (0.1)
Age		
0–19	88 891 (21.4)	6 (0.2)
20–29	66 519 (16.0)	14 (0.5)
30–39	88 967 (21.4)	44 (1.5)
40–49	62 116 (14.9)	103 (3.4)
50–59	46 612 (11.2)	247 (8.2)
60–69	30 577 (7.4)	562 (18.7)
70–79	18 763 (4.5)	862 (28.7)
80–89	10 158 (2.4)	909 (30.2)
90–99	2852 (0.7)	251 (8.3)
100+	171 (0.04)	9 (0.3)
Ethnicity		
Asian or Asian British	118 460 (28.5)	480 (16.0)
Black/African/Caribbean/Black British	20 877 (5.0)	71 (2.4)
Mixed/multiple ethnic groups	11 215 (2.7)	31 (1.0)
White	168 017 (40.4)	1958 (65.1)
Other ethnic group	14 836 (3.6)	53 (1.8)
Unknown	82 221 (19.8)	414 (13.8)

AF, atrial fibrillation.

### Outcome measures

#### AF prevalence and incidence

Annual AF prevalence recorded in Hounslow QOF data increased by 0.24 percentage points from 2011 to 2018, with most of this increase (0.20%) occurring between 2014 and 2018, this represents a 0.04% average annual increase in AF prevalence from 2014 to 2018 compared with 0.01% between 2011 and 2014.^17^

**Figure 1:**
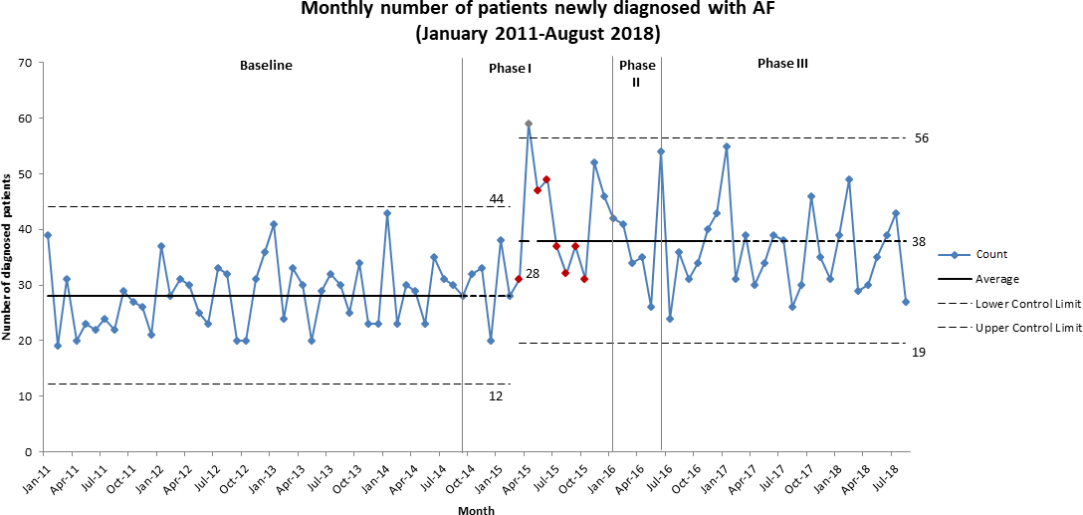
C-chart showing monthly number of patients newly diagnosed with AF, from January 2011 to August 2018. The increase from 28 patients newly diagnosed with AF each month in the baseline period to 38 each month is clearly seen in phase I of the intervention period. AF, atrial fibrillation.

In our ITS model, there was a statistically significant increase in the monthly rate of new AF diagnosis in the intervention period, with an increase of 27% (relative risk (RR) 1.27; 95% CI 1.05 to 1.52; p<0.01) (online supplementary file 4). Adjusting for seasonality did not alter this finding (RR: 1.24; 95% CI 1.04 to 1.48; p<0.01). Autocorrelation was assessed, and the lags were found not to have a significant effect. This increase is also seen in the raw number of patients newly diagnosed with AF, which increased from an average of 28 per month in the baseline period to 38 per month from March 2015, during phase I (figure 1).

10.1136/openhrt-2019-001086.supp4 Supplementary data

### Process measures

#### ECG screening

**Figure 2:**
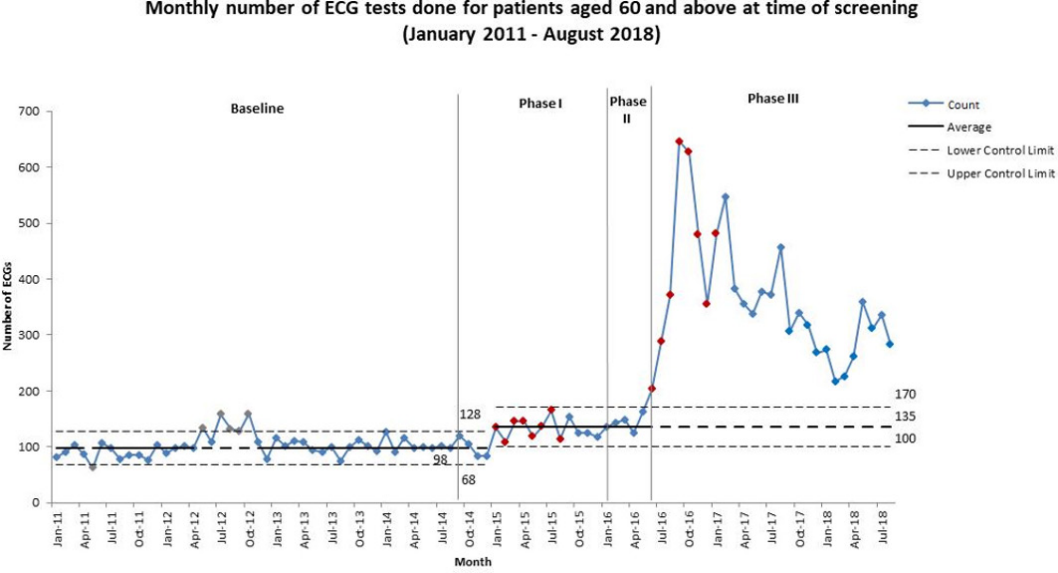
C-chart showing monthly number of ECG tests done for patients aged 60 and above at time of screening, from January 2011 to August 2018. The increase from 98 ECG tests done per month in the baseline period to 135 ECGs done is clearly seen in phase I of the intervention period. Although a further increase is observed in phase III of the intervention period, this increase was unstable, thus the control limits and centre line have not been revised.

The number of ECG tests done for patients aged 60 years and above increased from an average of 98 per month in the baseline period to 135 per month from January 2015 (phase I) (figure 2). A large transient increase was observed from June 2016 (phase II), but this did not persist.

#### CHA_2_DS_2_-VASc and HAS-BLED risk assessments

**Figure 3:**
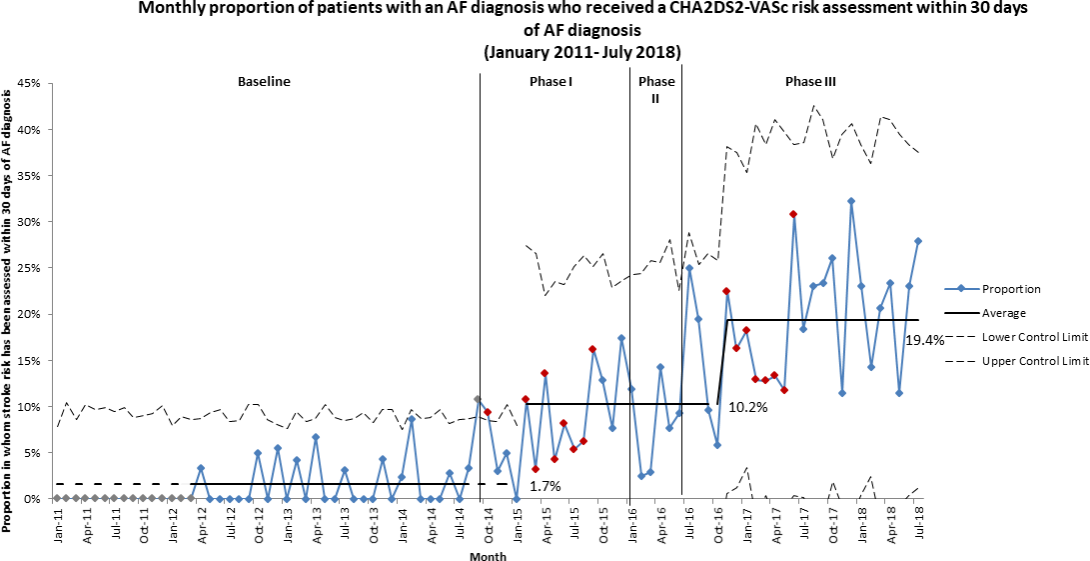
P-chart showing monthly proportion of patients with an AF diagnosis who received a CHA_2_DS_2_-VASc risk assessment within 30 days of AF diagnosis, from January 2011 to July 2018. The increase from 1.7% of patients with an AF diagnosis who received a CHA_2_DS_2_-VASc risk assessment within 30 days of AF diagnosis in the baseline period to 10% is clearly seen in phase I of the intervention period. A further increase to 19% is observed in phase III of the intervention period. AF, atrial fibrillation.

The monthly proportion of patients with an AF diagnosis who received a CHA_2_DS_2_-VASc risk assessment within 30 days of AF diagnosis increased from an average of 1.7% in the baseline period to 10% from February 2015 (phase I), and further rose to 19% from November 2016 (phase III) (figure 3). Similarly, increases were observed for the 90 days, 180 days and at any time from AF diagnosis measures (online supplementary file 5).

10.1136/openhrt-2019-001086.supp5 Supplementary data

**Figure 4:**
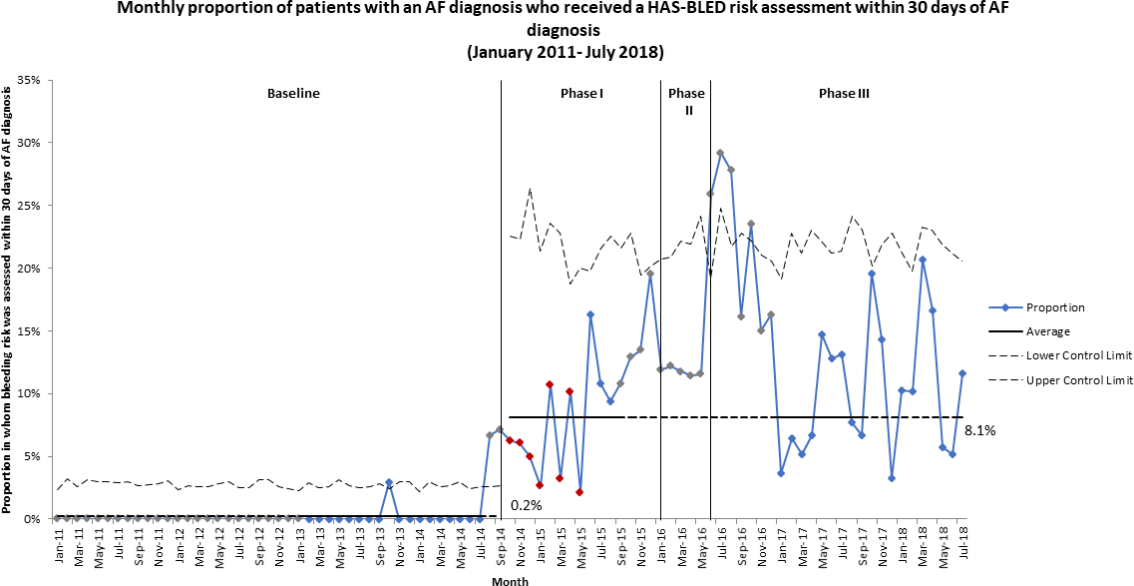
P-chart showing monthly proportion of patients with an AF diagnosis who received a HAS-BLED risk assessment within 30 days of AF diagnosis, from January 2011 to July 2018. The increase from 0.2% of patients with an AF diagnosis who received a HAS-BLED risk assessment within 30 days of AF diagnosis in the baseline period to 8.1% is clearly seen in phase I of the intervention period. AF, atrial fibrillation.

The monthly proportion of patients with an AF diagnosis who received a HAS-BLED risk assessment increased from an average of 0.2% in the baseline period to 8.1% from October 2014 (phase I) (figure 4). A transient increase was observed from September 2015 (phase I), but this did not persist. Similarly, increases were observed for the 90 days, 180 days and at any time from AF diagnosis measures (online supplementary file 5).

#### Anticoagulation and antiplatelet drug therapy

**Figure 5:**
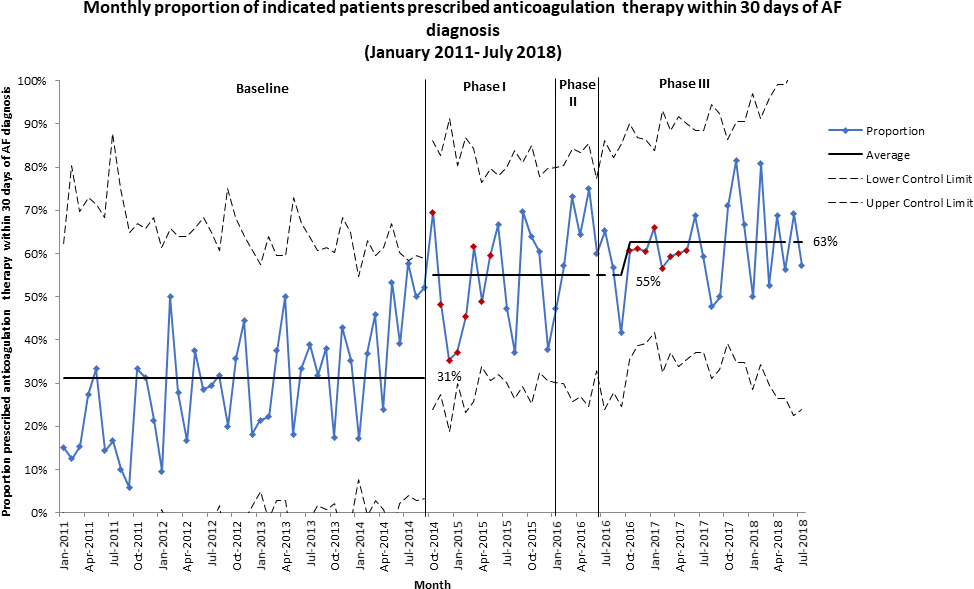
P-chart showing monthly proportion of indicated patients prescribed anticoagulation treatment within 30 days of AF diagnosis, from January 2011 to July 2018. The increase from 31% of indicated patients prescribed anticoagulation treatment within 30 days of AF diagnosis in the baseline period to 55% is clearly seen in phase I of the intervention period. A further increase to 63% is observed in phase III of the intervention period. AF, atrial fibrillation.

The monthly proportion of indicated patients prescribed anticoagulation drug therapy within 30 days of AF diagnosis increased from an average of 31% in the baseline period to 55% from October 2014 (phase I) (figure 5). A further increase to 63% is observed in phase III of the intervention period. Similarly, increases were observed for the 90 days, 180 days and at any time from AF diagnosis measures (online supplementary file 5).

**Figure 6:**
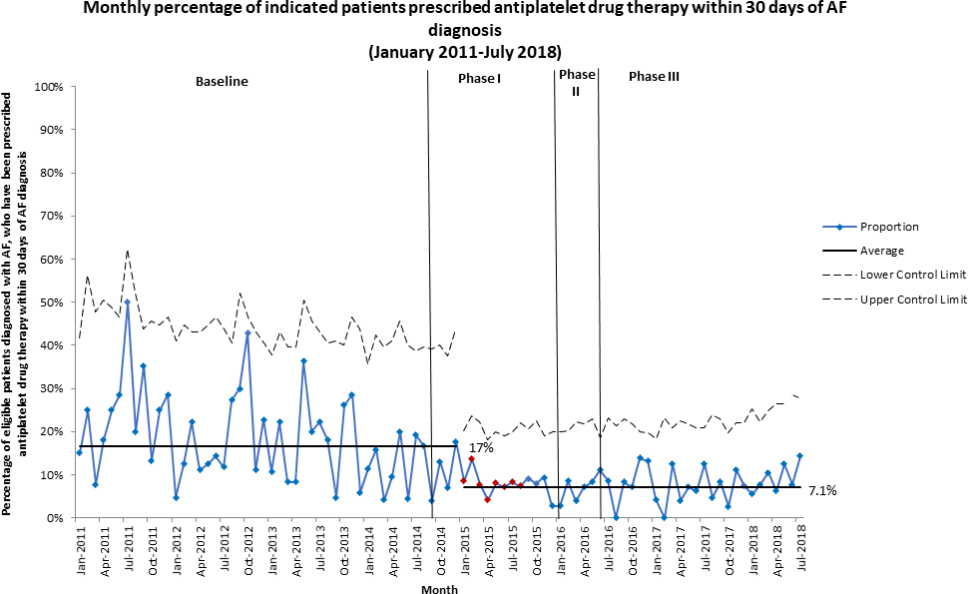
P-chart showing monthly proportion of indicated patients prescribed antiplatelet monotherapy within 30 days of AF diagnosis, from January 2011 to July 2018. The decrease from 17% of indicated patients who were prescribed antiplatelet monotherapy within 30 days of AF diagnosis in the baseline period to 7.1% is clearly seen in phase I of the intervention period. AF, atrial fibrillation.

Conversely, the monthly proportion of indicated patients prescribed antiplatelet monotherapy within 30 days of AF diagnosis decreased from an average of 17% in the baseline period to 7.1% from January 2015 (phase I) (figure 6). Similarly, decreases were observed for the 90 days, 180 days and at any time from AF diagnosis measures (online supplementary file 5).

### Balancing measures

#### CHA_2_DS_2_-VASc and HAS-BLED risk scores

The monthly average CHA_2_DS_2_-VASc score in the baseline period was 3.4 and this remained the same throughout the intervention period (online supplementary file 6). Similarly, average HAS-BLED risk scores in the intervention period did not change from the baseline average of 1.6 (online supplementary file 7).

10.1136/openhrt-2019-001086.supp6 Supplementary data

10.1136/openhrt-2019-001086.supp7 Supplementary data

Indicated patients who have a HAS-BLED score are more likely to be anticoagulated than those who do not have a HAS-BLED score (p<0.001).

## Discussion

### Principal findings

Between baseline and intervention periods, the number of ECG tests conducted per month for patients aged 60 and above increased. Diagnosed incidence and prevalence of AF also increased, although prevalence remained lower than the level expected for the population. Within those newly diagnosed with AF, the proportion who received timely CHA_2_DS_2_-VASc and HAS-BLED risk assessments increased, and risk profiles did not change. A greater proportion of patients indicated for anticoagulation received appropriate prescriptions, while prescription of antiplatelet monotherapy decreased. Despite these promising improvements in measures related to management of AF, there remains room for improvement in provision of timely care. This is supported by the fact that, on lifting the time limit (ie, 30/90/180 days from AF diagnosis) for these measures, the average proportion of patients receiving best practice elements of care increases (online supplementary file 5).

### ECG screening

Increases in ECG screening suggest that a combination of HCP education, availability of handheld ECG devices, an AF at-risk register and an AF clinical template with a linked patient status alert is a viable means of increasing opportunistic screening in primary care. However, increases were unstable, and the possible decline from September 2017 may indicate that further HCP engagement is necessary, or that following an initial increase in diagnoses, the remaining undiagnosed population is still not being reached.

### AF prevalence and incidence

AF prevalence and incidence increased during the intervention period. Demographic factors remained relatively unchanged between 2011 and 2017, indicating that the increases were not due to a changing proportion of frail elderly. There was a steady population increase in Hounslow between 2011 and 2018 in contrast with the step change in new AF diagnoses. This suggests that the increase in AF diagnosis rate is not simply a reflection of increasing population. Although we cannot assume causality, it is possible that HCP education may have contributed to the increase in the number and rate of new AF diagnosis. However, increased AF prevalence and incidence may be associated with other secular trends such as national focus on AF and anticoagulation (eg, AF awareness week) or introduction of the NICE guidelines on AF diagnosis and management in June 2014. After the initial increase of new AF diagnosis in March 2015, there were no further increases observed. This indicates that the distribution of handheld ECG devices from June 2016 onwards and the accompanying increase in screening did not result in higher case detection. A possible reason is that at-risk but ultimately healthy patients were being screened—for example, there may be participation bias where population characteristics differ between general practice attenders and non-attenders, in terms of health-seeking behaviours and health status.

### CHA_2_DS_2_-VASc and HAS-BLED risk assessments

Improvements in stroke and bleeding risk assessments for patients with an AF diagnosis occurred during the intervention period. It is plausible that increases in risk assessments after June 2014 are associated with the updated NICE guidelines, at least at the start of the intervention period. Further increases may be due to the QI initiative. Nonetheless, improved timeliness of risk assessments over time is indicative of increased clinical activity towards mitigating AF-related stroke risk.

Although guidelines state that bleeding risk should be taken into consideration for patients diagnosed with AF to identify and treat risk factors, the proportion of patients in whom bleeding risk was assessed was lower than that of stroke risk assessments. Despite increases in bleeding risk assessments during QI initiative’s active funding period (October 2015 to March 2017), these increases were not sustained. The lower proportion of bleeding risk assessments could be due to these risk assessments historically not being done in routine practice for patients diagnosed with AF^27^ or may be because bleeding risk assessments unlike stroke risk assessments are not nationally incentivised in the QOF. During the initiative, the QI team were careful to emphasise the 2016 ESC guideline position: ‘A high bleeding risk score should generally not result in withholding oral anticoagulation. Rather, bleeding risk factors should be identified and treatable factors corrected.’^4^

### Anticoagulation drug therapy and antiplatelet monotherapy

Anticoagulation prescription in patients for whom it was indicated increased during the intervention period, with a decrease in antiplatelet monotherapy. Improvements in these measures may be due to the QI initiative but as previously mentioned causality cannot be attributed on the basis of this study. Despite these improvements, the proportion of indicated patients receiving anticoagulants within 30 days is still quite low. Reasons for this could include retrospective or delayed data recording in SystmOne or patient choice. It seems unlikely that bleeding risk played a major role in this, however, based on the result that indicated patients scored for bleeding are more likely to be prescribed anticoagulation than those not scored.

A small proportion of patients with an AF diagnosis are still on aspirin monotherapy despite guideline recommendations against its prescription for stroke prevention in patients diagnosed with AF.^10^ This may be due to patient preferences or aspirin monotherapy prescription for another comorbidity, but also suggests that further work needs to be undertaken around this.

### CHA_2_DS_2_-VASc and HAS-BLED risk scores

Stable CHA_2_DS_2_-VASc and HAS-BLED risk scores in the intervention period means that the increased screening did not lead to detection of AF in patients with a different risk profile when compared with the baseline period.

### Comparison with existing literature

Findings from this study concur with previous studies showing HCP education, clinical decision support tools and screening can contribute to improved AF diagnosis and management.^12 13 28–30^ Studies have tested the feasibility and accuracy of using handheld ECG devices in AF screening programmes in different settings, by various users, for both systematic and opportunistic screening approaches.^10 31–33^ All report acceptable rates of newly diagnosed AF and positive perceptions towards the technology. This research goes one step further focusing on implementation of this device in routine care.

### Strengths and limitations

Use of routinely collected primary care data from all but one Hounslow general practice minimises selection bias, and including both outcome and process measures provide a thorough assessment of AF diagnosis and management pathways.

It is difficult to know if improvements were associated with the interventions, wider secular trends, or unmeasured confounders such as guidelines, reimbursement, and so on, because there is no control group. The QI initiative may have only temporarily changed GPs’ behaviours, as suggested by the decline in some measures towards the end of the study. Retrospective data on the AF at-risk population were unavailable, hence ECG tests done for patients aged 60 years and above were used as a proxy measure for ECGs done within the AF at-risk population. Using the available data, it was not possible to distinguish between ECG tests and AF diagnoses resulting directly from use of the KardiaMobile device, and those resulting from traditional ECG screening. AF diagnoses were only available from 2011 onwards, hence, there may be patients with unknown AF within the study population. The proportion of CHA_2_DS_2_-VASc risk assessments in the baseline period may not be a true representation of stroke risk assessments done, as the older, less comprehensive CHADS_2_ risk assessment tool was used prior to NICE recommending use of the CHA_2_DS_2_-VASc risk assessment tool^34^ and also patients with an AF diagnosis may still be prescribed anticoagulants at a clinician’s discretion without having done a CHA_2_DS_2_-VAS_C_ risk assessment or the risk assessment may be done but not recorded. Finally, population and practice characteristics of Hounslow could limit generalisation of the findings.

### Implications for future research

Further research should focus on the reasons behind the remaining gaps between expected and observed prevalence, including developing more subtle prediction models and understanding prevalence in subpopulations. It is not clear to what extent the interventions deployed in the Hounslow QI initiative are suitable to reveal further undiagnosed prevalence, and further improve risk scoring and anticoagulation, or whether additional interventions may be required to achieve this. The long-term impact of this initiative is not clear from the data available for this study, future research should look at hospital admissions for stroke and bleeding with history of AF, as well as HCP and patient perceptions of the QI initiative, as well as barriers and facilitators to sustaining the observed gains.

## Conclusion

Using electronic health record data, this study found that implementation of synergistic evidence-based interventions in a QI initiative coincided with improvements in AF diagnosis and management in 47 general practices in NWL. It has highlighted the disparity between quality of care as measured by QOF data and more granular analysis including timeliness of patient care. Other healthcare areas with perceived underdetection of AF should consider similar interventions and methodology to improve AF diagnosis and management.
