# Supplementary material for: Improving the quality of care for patients with or at risk of atrial fibrillation: an improvement initiative in UK general practices
Source: Open Heart. 2019 Oct 15;6(2):e001086. doi: 10.1136/openhrt-2019-001086 (PMC6802985; doi:10.1136/openhrt-2019-001086)
Supplement: Supplementary data [file openhrt-2019-001086supp002.pdf]

Statistical Process Control (SPC) charts are a graphical family of techniques designed for analysing data over time. SPC uses a number of ‘rules’ to determine whether a process exhibits unusual variation (special causes), or if fluctuations observed are simply down to chance. In this study, we use C-, P- and X-bar-S- charts for count, proportion and continuous measures respectively. In each case, a centre line shows the mean, and a pair of control limits, set at 3 standard deviations from the centre line, indicate the usual range of variation for the measure.

We use the following established set of rules for detecting special causes:

- Rule 1: one or more data points outside of the control limits
- Rule 2: eight or more points consecutively above or below the centre line (a shift)
- Rule 3: six or more all increasing or decreasing values (a trend)
- Rule 4: two out of three consecutive points are near (in the outer one-third of the chart) a control limit, and
- Rule 5: 15 consecutive points within one standard deviation of the centre line.

Special cause variation detected through these rules is highlighted in red. If a shift is detected in the measure, and there is sufficient data to form a new stable centre line and control limits, then new limits are calculated and displayed for the new period following the shift. Data points highlighted in grey represent special cause variation excluded from the centre line and control limits, as they do not reflect typical behaviour for this period. This process is known as ‘Ghosting’.
