# Supplementary figures and images for: Improving the quality of care for patients with or at risk of atrial fibrillation: an improvement initiative in UK general practices
Source: Open Heart. 2019 Oct 15;6(2):e001086. doi: 10.1136/openhrt-2019-001086 (PMC6802985; doi:10.1136/openhrt-2019-001086)

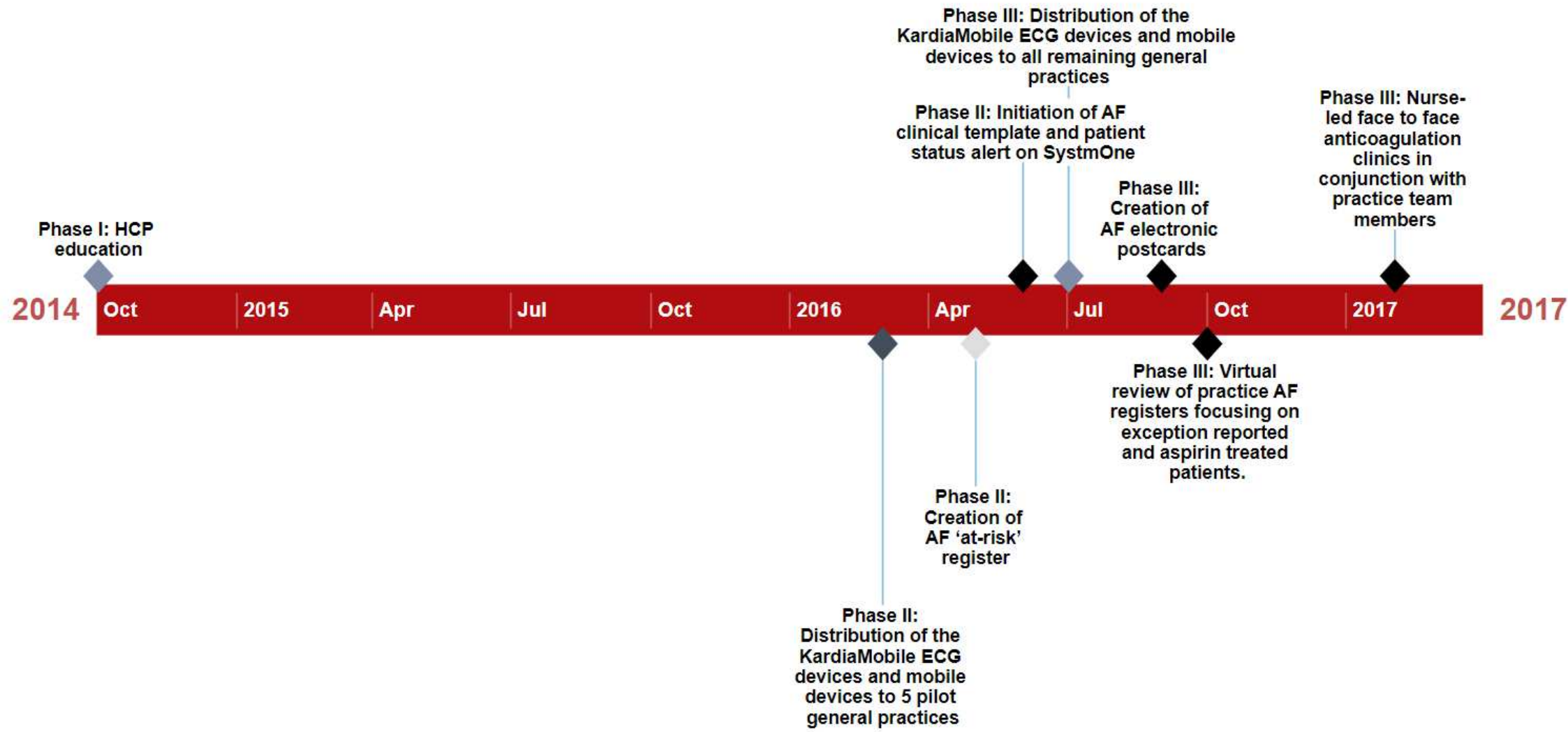

Supplement: Supplementary data [file openhrt-2019-001086supp001.pdf]

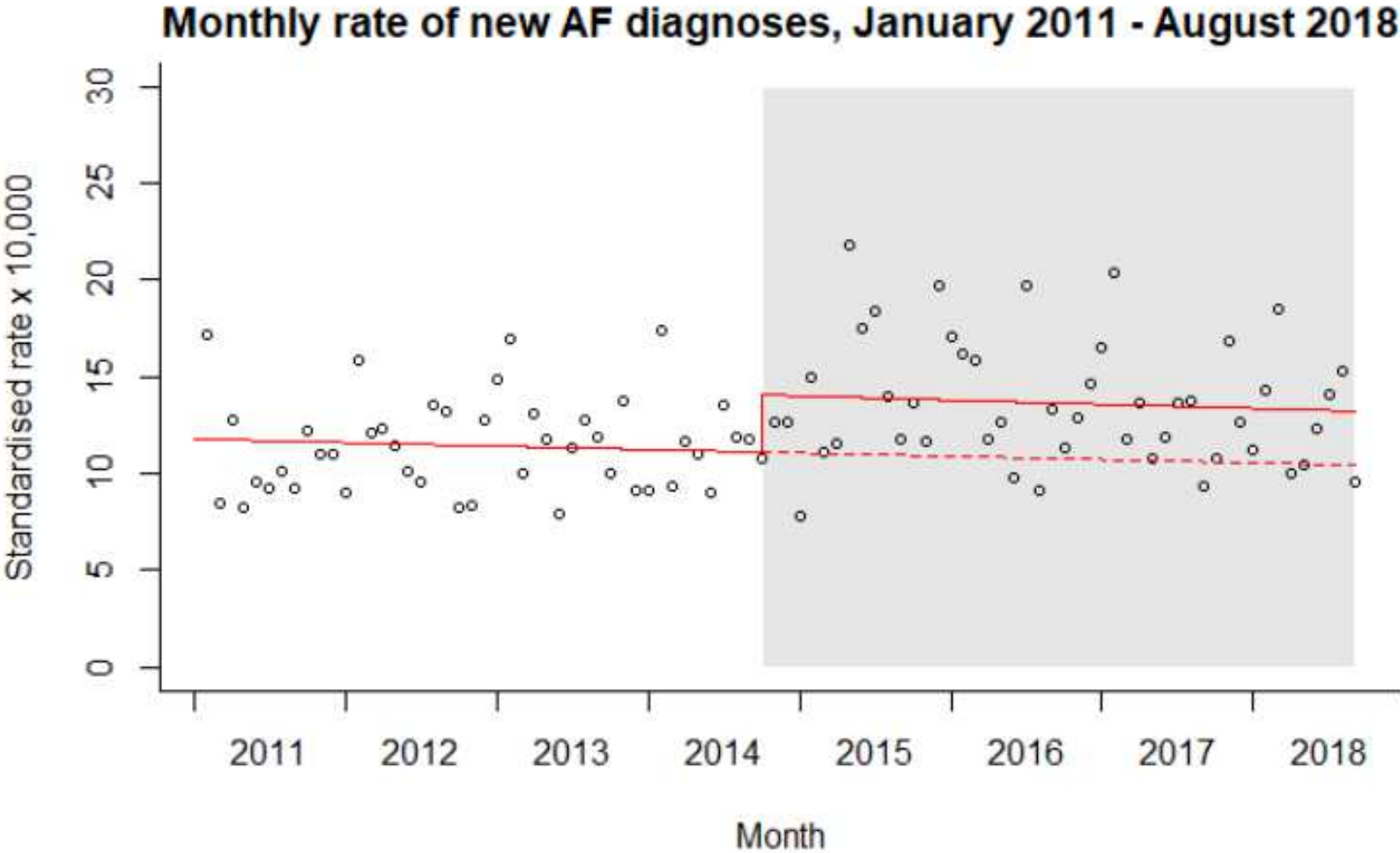

Supplement: Supplementary data [file openhrt-2019-001086supp004.pdf]

Monthly average CHA2DS2-VASc score (January 2011 – August 2018)

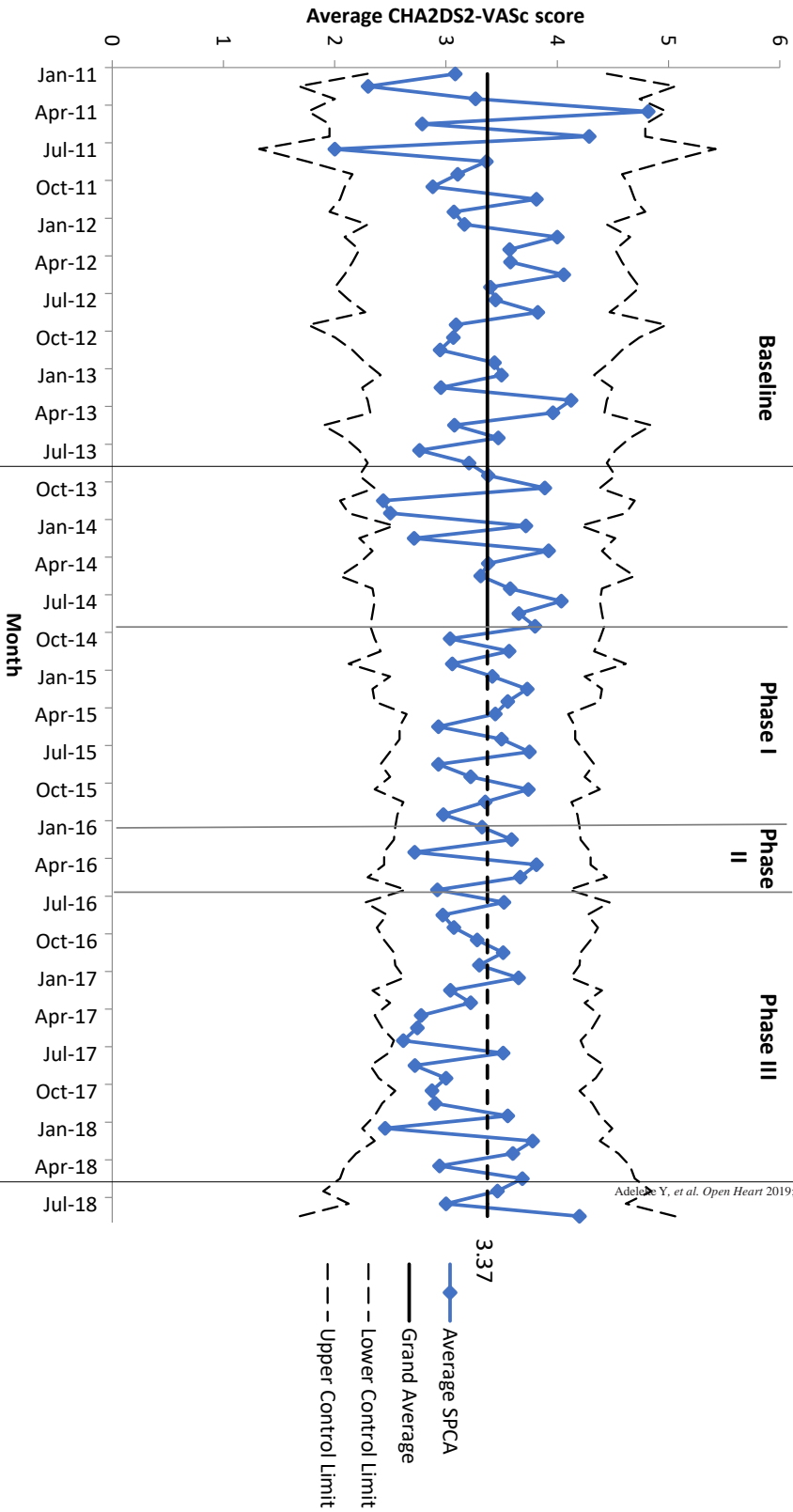

Supplement: Supplementary data [file openhrt-2019-001086supp006.pdf]

Monthly average HAS-BLED score (January 2011 – August 2018)

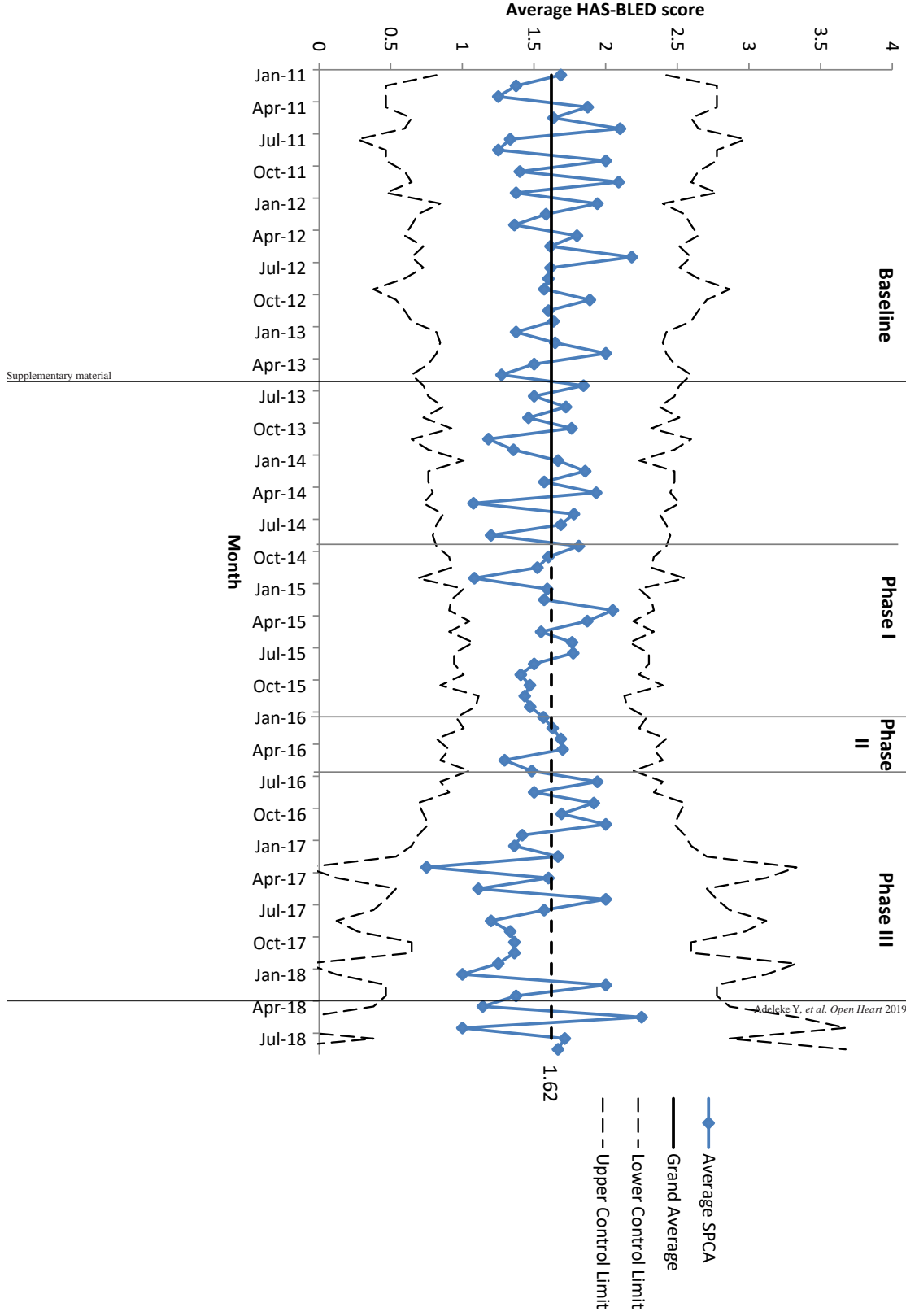

Supplement: Supplementary data [file openhrt-2019-001086supp007.pdf]
