# Supplementary material for: Improving the quality of care for patients with or at risk of atrial fibrillation: an improvement initiative in UK general practices
Source: Open Heart. 2019 Oct 15;6(2):e001086. doi: 10.1136/openhrt-2019-001086 (PMC6802985; doi:10.1136/openhrt-2019-001086)
Supplement: Supplementary data [file openhrt-2019-001086supp003.pdf]

Characteristic	Population with active registration status as at 31st December, 2011 (%)	Population with active registration status as at 31st December, 2014 (%)	Population with active registration status as at 31st December, 2017 (%)
**Sex**			
Female	64701 (40.7)	81875 (41.9)	112589 (43.6)
Male	94186 (59.3)	113591 (58.1)	145770 (56.4)
Other/Unknown	6 (0.0)	7 (0.0)	12 (0.0)
**Age**			
0-19	32632 (20.5)	40156 (20.5)	54482 (21.1)
20-29	20381 (12.8)	24442 (12.5)	33662 (13.0)
30-39	29505 (18.6)	36630 (18.7)	50598 (19.6)
40-49	30515 (19.2)	35214 (18.0)	43096 (16.7)
50-59	22174 (14.0)	27688 (14.2)	34953 (13.5)
60-69	14765 (9.3)	18391 (9.4)	22622 (8.8)
70-79	6956 (4.4)	9425 (4.8)	12954 (5.0)
80-89	1802 (1.1)	3135 (1.6)	5100 (2.0)
90-99	147 (0.1)	363 (0.2)	852 (0.3)
100+	16 (0.0)	29 (0.0)	52 (0.0)
**Ethnicity**			
Asian or Asian British	38067 (24.0)	50393 (25.8)	71751 (27.8)
Black/African/Caribbean/ Black British	7889 (5.0)	10086 (5.2)	13296 (5.1)
Mixed/Multiple Ethnic Groups	3595 (2.3)	4945 (2.5)	7151 (2.8)
White	62737 (39.5)	78266 (40.0)	105135 (40.7)
Other Ethnic Group	4410 (2.8)	5832 (3.0)	9146 (3.5)
Unknown	42195 (26.6)	45951 (23.5)	518892 (20.1)
