# Supplementary material for: Improving the quality of care for patients with or at risk of atrial fibrillation: an improvement initiative in UK general practices
Source: Open Heart. 2019 Oct 15;6(2):e001086. doi: 10.1136/openhrt-2019-001086 (PMC6802985; doi:10.1136/openhrt-2019-001086)
Supplement: Supplementary data [file openhrt-2019-001086supp005.pdf]

Measure	Time period	30 days of AF diagnosis (%)	90 days of AF diagnosis	180 days of AF diagnosis	At any time point after AF diagnosis
**Monthly proportion of patients with an AF diagnosis who received a CHA2DS2-VASc risk assessment**	Baseline	1.7	3.0	3.9	76.3
	Phase I-II	10.2	15.4	27.1	92.0
	Phase III	19.4	33.3	27.1	92.0
**Monthly proportion of patients with an AF diagnosis who received a HAS-BLED risk assessment**	Baseline	0.2	1.0	1.2	40.4
	Phase I - II	8.1	15.2	14.2	62.9
	Phase III	8.1	15.2	14.2	20.5
**Monthly proportion of indicated patients prescribed anticoagulation drug therapy**	Baseline	31.2	48.3	57.7	83.2
	Phase I - II	55.1	76.7	83.2	91.0
	Phase III	62.8	76.7	83.2	91.0
**Monthly proportion of indicated patients prescribed antiplatelet monotherapy**	Baseline	16.7	17.6	18.8	23.2
	Phase I - III	7.1	8.6	8.7	9.3
